# Enhanced Agronomic Efficiency Using a New Controlled-Released, Polymeric-Coated Nitrogen Fertilizer in Rice

**DOI:** 10.3390/plants9091183

**Published:** 2020-09-11

**Authors:** Ricardo Gil-Ortiz, Miguel Ángel Naranjo, Antonio Ruiz-Navarro, Sergio Atares, Carlos García, Lincoln Zotarelli, Alberto San Bautista, Oscar Vicente

**Affiliations:** 1 Institute for Plant Molecular and Cell Biology (UPV-CSIC), Universitat Politècnica de València, 46022 Valencia, Spain; mnaranjo@ibmcp.upv.es; 2 Fertinagro Biotech S.L., Polígono de la Paz, C/ Berlín s/n, 44195 Teruel, Spain; sergio.atares@tervalis.com; 3 Centre for Soil and Applied Biology Science of Segura (CEBAS-CSIC), Espinardo University Campus, 30100 Murcia, Spain; ruiznavarro@cebas.csic.es (A.R.-N.); cgarizq@cebas.csic.es (C.G.); 4 Horticultural Sciences Department, University of Florida, Gainesville, FL 32611, USA; lzota@ufl.edu; 5 Plant Production Department, School of Agricultural Engineering and Environment, Universitat Politècnica de València, 46022 Valencia, Spain; asanbau@prv.upv.es; 6 Institute for the Conservation and Improvement of Valencian Agrodiversity (COMAV), Universitat Politècnica de València, 46022 Valencia, Spain; ovicente@upvnet.upv.es

**Keywords:** controlled-release fertilizer, humic acid, lignosulfonate, natural polymers, *Oryza sativa*

## Abstract

Fertilizer-use efficiency is one of the most critical concerns in rice cultivation to reduce N losses, increase yields, and improve crop management. The effects of a new polymeric-coated controlled-release fertilizer (CRF) were compared to those of other slow-release and traditional fertilizers in a microscale experiment, which was carried out in cuvettes under partly controlled ambient conditions, and a large-scale field experiment. To evaluate the fertilizer’s efficiency, nitrogen and water-use efficiency were calculated using the measurement of different photosynthetic and crop yield parameters. Improved responses regarding some of the analyzed physiological and growth parameters were observed for those plants fertilized with the new CRF. In the microscale experiment, significantly increased yields (ca. 35%) were produced in the plants treated with CRF as compared to traditional fertilizer. These results were in accordance with ca. 24% significant increased levels of N in leaves of CRF-treated plants, besides increased P, Fe, Mn, and cytokinin contents. At the field scale, similar yields were obtained with the slow-release or traditional fertilizers and CRF at a 20% reduced N dose. The new controlled-release fertilizer is a urea-based fertilizer coated with lignosulfonates, which is cheaply produced from the waste of pulp and wood industries, containing humic acids as biostimulants. In conclusion, CRF is recommended to facilitate rice crop management and to reduce contamination, as it can be formulated with lower N doses and because it is ecological manufacturing.

## 1. Introduction

According to the Food and Agriculture Organization of the United Nations (FAO), rice is the second most important cereal worldwide in terms of production, after maize, and the third in terms of cultivated area, after wheat and maize [1]. Rice production in 2017/2018 increased by 1.6% with respect to the previous year, which left global production at a new maximum of 782 million tons [1]. Worldwide rice milled production reached 496 million tons in 2018/2019, with consumption increasing by 2% in 2019/2020. Despite the global production increase, the world population is expected to grow proportionally and will reach ca. 10 × 10^9^ people by 2050. That is why one of the main challenges of current agriculture is to search for new methods to increase the yield and quality of crops through sustainable agriculture [2,3]. Improving nutrient-use efficiency is one of the major objectives when establishing fertilization programs in cereals, because increased productivity and reduced contamination are achieved [4,5]. Efficient nutrient use in flood-irrigated rice plots depends on different factors, such as supplying essential nutrients at adequate rates, sources, application methods, and application times [6,7,8]. Nitrogen is the primary nutritional element that determines the plant’s growth and the yield of most crops, and it is highly mobile in soil [9]. Nitrogen is used in metabolic processes, including the production of nucleic acids, proteins, and cofactors, and it is also used in the synthesis of signaling and storage molecules [10]. Ammonium (NH_4_^+^) and nitrate (NO_3_^−^) can be easily removed from soils by drainage, denitrification (N_2_O), volatilization (NH_3_), or surface runoff [11,12,13]. The majority of the world rice cultivation is performed in irrigated-lowland systems under anaerobic soil conditions, and NH_3_ volatilization and denitrification are the main ways of losing N [11,14,15]. Using NO_3_^−^ fertilizers for pre-flood N application is not recommended, because NO_3_-N is subjected to denitrification after flooding, and rice seedlings are sensitive to NO_3_^−^ salts [9]. Currently, the most widely used fertilizers in rice cultivation remain enriched urea and ammonium sulfate, as they are the most effective in flooded systems and offer a moderate benefit/cost ratio [11,16]. However, it has been reported that ammonia volatilization may even cause losses of up to 60% of the urea–N fertilizers applied to flooded soils [17]. Traditional urea fertilizers usually have poor N-release kinetics, so N losses are very high after they have been applied [9,18]. Based on European legal framework regulations, significant efforts have been made to develop new fertilizers that reduce the N losses observed from traditional fertilizers [19,20].

Nitrogen fertilizers with enhanced efficiency, such as slow- or controlled-release fertilizers (SCRFs), are an alternative to achieving higher agronomic efficiency than traditional fertilizers [17,21,22]. In fact, SCRFs usually have nutrient emission kinetics closer to plant requirements and significantly reduce contamination [23,24]. Unlike SRFs, controlled-release fertilizers (CRFs) are less influenced by soil temperature or texture, and they are not so dependent on soil microbiology [18,25,26]. A single CRF base application to soil during crop establishment usually suffices to cover the plant’s nutrient requirements, reducing cultivation costs [18,27,28]. Although major improvements have been achieved, the majority of CRFs developed to date have been manufactured from plastic polymers, which are barely degradable and incur high production costs [23,29]. Different synthetic CRFs have been developed in the past, such as urea formaldehyde, isobutylidene diurea, crotonylidene diurea, or sulfur-coated urea fertilizers [30]. To cite some examples in rice, urea formaldehyde, sulfur, and polymer-coated urea have been applied successfully to increase yields and quality [2,31]. Polyoleofin resin-coated NPK granules enriched with micronutrients control iron deficiency by co-situs applications together with seeds or seedlings [32]. Granulated urea-intercalated kaolinite-coated with water-based epoxy resin diminished N emission in flooded rice crops by 3-fold and increase yields by around 2-fold compared to conventional urea [17]. Reductions in N_2_O emission levels have also been found when using thermoplastic resin-coated urea [33]. In this context, the use of lignin-based, water-soluble synthetic products or natural polymers represents an alternative to these problems posed by CRFs produced from plastic polymers, as they can be obtained in large quantities and cheaply from the waste generated in the paper and wood industries [34,35]. Presently, SCRFs have been limited mainly to horticultural and ornamental crops, and they are not well established in extensive cropping. Thus, more research is necessary to produce cheaper and environmentally friendlier fertilizers [36,37,38]. The objective of this research was to compare the efficacy of a new ecological controlled-release coated urea fertilizer with traditional ones in rice, regarding physiological responses and grain yield and quality. The novelty of this research lies in combining a urea-based CRF coated with a natural polymer, generated as a by-product of wood pulp production, with a urease inhibitor and natural biostimulants.

## 2. Results

### 2.1. Plant Growth, Leaf Greenness, and Effective Quantum Yield of Photosystem II

**Figure 1:**
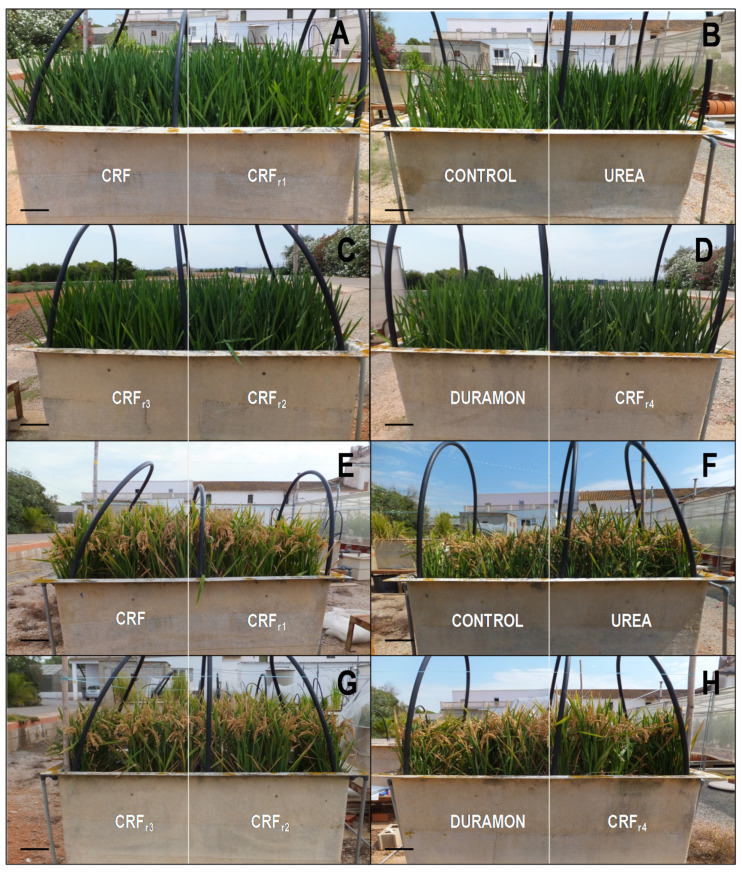
Comparison of rice growth responses, on the microscale experiment, to the applied CRF and their reductions from CRF_r1_ to CRF_r4_, DURAMON^®^, UREA, and the CONTROL (no fertilizer applied) in the phenological stage of booting (**A**–**D**) and at the end of culture (**E**,**F**). Treatments from left to right: CRF, CRF_r1_ (**A**,**E**); CONTROL, NSA (**B**,**F**); CRF_r3_, CRF_r2_ (**C**,**G**); DURAMON^®^, CRF_r4_ (**D**,**H**). Bars correspond to 10 cm.

The responses of the growth parameters measured by non-destructive techniques are shown in Figure 1 and Table 1. No significant differences were found between the different fertilizer treatments applied in the phenological booting stage for total fresh weight, total length, primary stem length, tillers number, and total foliar area. Nor was there any clear tendency by the significant differences observed between CRF and CRF_r1_ to CRF_r4_ in respect to the rest of the treatments for the measured parameters of dry mass (DW) (aerial part) (%) and leaf weight (g); furthermore, no significance was observed for leaf RWC between the applied treatments. For the photosynthetic parameters responses of ΦPSII, no significant differences were detected between the distinct fertilizer treatments, although higher levels leaf greenness content were produced in the CRF treatments and their reductions to CRF_r3_ and DURAMON^®^ compared to CRF_r4_ and UREA.

### 2.2. Gas Exchange Analysis

**Figure 2:**
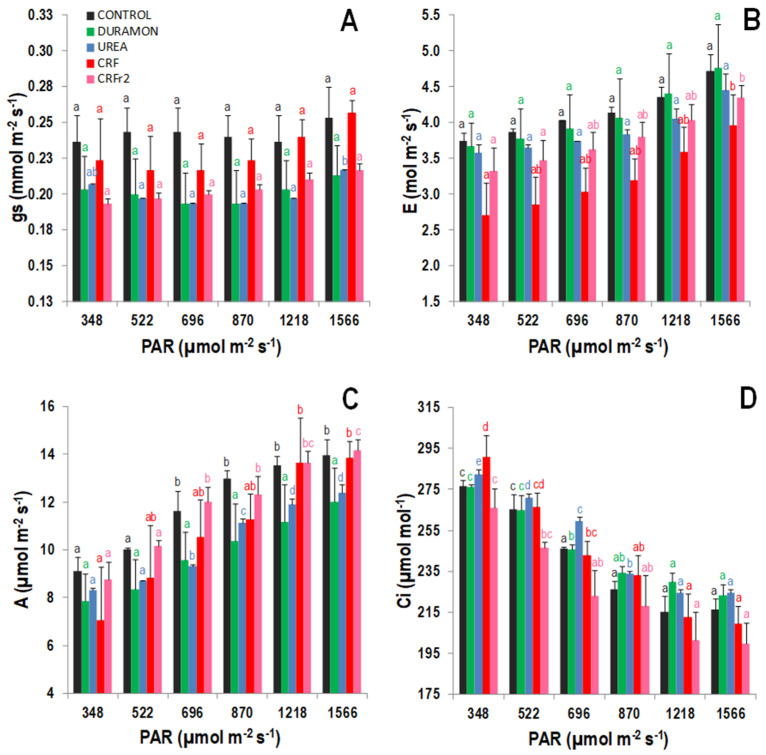
Gas exchange responses, on the microscale experiment, to the different photosynthetically active radiation (PAR) rates of *Oryza sativa* treated with fertilizers CRF, CRF_r2_, DURAMON^®^, UREA, and the CONTROL (no fertilizer applied) in the phenological booting stage. (**A**) Stomatal conductance, (**B**) transpiration rate, (**C**) photosynthetic rate, and (**D**) substomatal CO_2_ concentration. Values represent means ± SD (*n* = 5). Different letters (same color) indicate statistically significant differences between different PAR levels for each treatment (ANOVA, *p* < 0.05).

**Figure 3:**
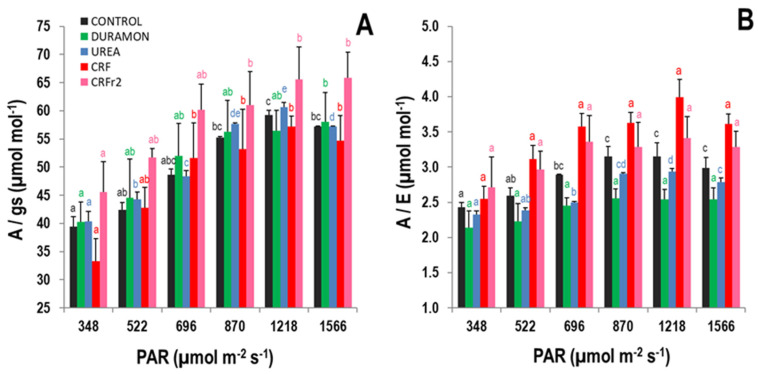
Water-use efficiency responses, on the microscale experiment, to the different photosynthetically active radiation (PAR) rates of *Oryza sativa* treated with fertilizers CRF, CRF_r2_, DURAMON^®^, UREA, and the CONTROL (no fertilizer applied), in the phenological booting stage. (**A**) Water-use efficiency and (**B**) intrinsic water-use efficiency. Values represent means ± SD (*n* = 5). Different letters (same color) indicate statistically significant differences between different PAR levels for each treatment (ANOVA, *p* < 0.05).

The responses to the analyzed gas exchange parameters are shown in Figure 2. Increased *A* and *E* values were observed as rising photosynthetically active radiations (PAR) levels were applied from 348 to 1566 µmol m^−2^ s^−1^. Under the same conditions, the *gs* levels showed slight increases and *Ci* tendencies diminished. Nonetheless, higher *A* and *gs* levels were produced in the plants treated with CRF and CRF_r2_ compared to urea and DURAMON^®^, and their levels in the CONTROL plants were similar to CRF, with no significant differences between them. On average, the *gs* and *E* levels were lower in the plants treated with CRFs compared to urea and DURAMON^®^. Significant differences were observed only in the *Ci* values at lower PAR of 348 and 522 µmol m^−2^ s^−1^. At 348 µmol m^−2^ s^−1^ PAR, the *Ci* levels in the plants treated with CRF were significantly higher than for CRF_r2_, but under 522 µmol m^−2^ s^−1^ PAR, the values for the UREA-treated plants were significantly higher than for CRF_r2_. In any case, these differences were not significant at upper PAR levels. On average, the *A/gs* levels at 1566 µmol m^−2^ s^−1^ of the CRF_r2_-fertilized plants were 1.2-fold higher than for the other treatments (Figure 3A). Maximum differences of the *A/E* levels took place at 1218 µmol m^−2^ s^−1^ PAR, and the CRF levels were 1.4- and 1.6-fold significantly higher as compared to DURAMON^®^ and UREA, respectively (Figure 3B). Significant differences between CRF and UREA also occurred at 1566 µmol m^−2^ s^−1^ PAR, and significant differences for DURAMON^®^ occurred from 696 to 1566 µmol m^−2^ s^−1^ PAR.

### 2.3. Foliar Nutrient Content

**Figure 4:**
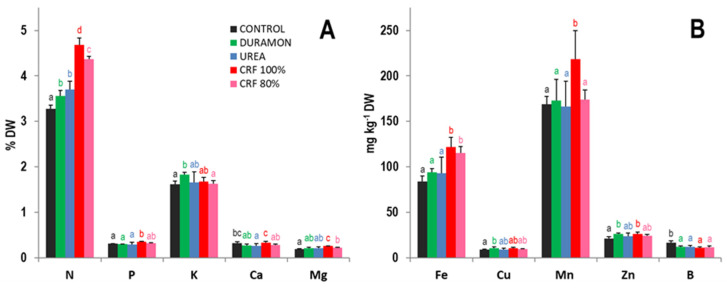
Macro- (**A**) and micronutrient (**B**) foliar content, on the microscale experiment, in rice fertilized with CRF, CRF_r2_, DURAMON^®^, UREA, and CONTROL (no fertilizer applied), in the phenological booting stage. The macronutrients results (N, P, K, Ca, Mg, and S) are expressed as percentage (*w/w*) of leaf dry mass (DW), and micronutrients as mg kg^-1^ leaf DW. Values are means ± SD, *n* = 4 (pools of ≥ 10 leaves). Different letters for a specific macro- or micronutrient in each panel indicate statistically significant differences between treatments (ANOVA, *p* < 0.05).

The results of the macro- and micronutrient contents in flag leaves in the phenological stage of panicle swelling are shown in Figure 4. The CRF treatment was the most effective at increasing the levels of nutrients in leaves. The N content was 1.3-fold significantly higher in the CRF-fertilized rice versus DURAMON^®^ and UREA applied at the same dose (Figure 4A). Similarly, P and Mg levels significantly increased by 1.1- and 1.2-fold, respectively. In general, no significant differences were found for K and Ca levels between the applied treatments. Concerning micronutrient content (Figure 4B), Fe and Mn levels significantly increased by 1.3-fold for the CRF treatment compared to DURAMON^®^ and UREA. No differences were found for the Cu, Zn, and B levels between the different fertilizer treatments.

### 2.4. Hormone Activity

As shown in Table 2, the indoleacetic acid (*IAA*) content did not differ significantly between the different treatments, including the CONTROL. The jasmonic acid (*JA*) levels were significantly higher for the UREA-treated plants compared to the other treatments, but they were not significantly different between CRF and the CONTROL. The salicylic acid (*SA*) content was significantly lower in CRF than in the other treatments and the CONTROL. The abscisic acid (*ABA*) levels were significantly higher in the CONTROL than in CRF- and DURAMON^®^-treated plants, with maximum 1.6-fold differences. The cytokinins (*CK*) levels were significantly higher in the CRF-treated plants compared to the other applied treatments. The isopentenyl adenine (*iP*) content for CRF significantly increased by 9-, 6- and 9-fold compared to the DURAMON®, UREA, and the CONTROL, respectively. Similarly, significant increases of 4.7-, 2-, and 2.4-fold were observed in tZ content, respectively. However, dihydrozeatin (*DHZ*) was not detected in any of the studied samples.

### 2.5. Growth, Yield, and Cereal Grain Composition

The results of growth and yield parameters obtained in the microscale experiment at the end of culture are shown in Table 3. No significant differences were found among the fertilizer treatments at the maximum doses applied for total fresh and dry weights, tillers number, and panicle length. The primary stem lengths in the plants fertilized with CRF and CRF_r1_ were 1.1-fold significantly higher compared to DURAMON^®^ and UREA. Higher yields were obtained in the CRF-treated rice, with total panicle weight being 1.4- and 1.6-fold significantly higher than for DURAMON^®^ and UREA, respectively. These differences were mainly caused by a significant increase in grain number of 1.2- and 1.5-fold, respectively. Panicle number was significantly smaller in the plants treated with UREA than for the other fertilizer treatments, with a maximum 1.3-fold difference versus CRF_r1_. Despite these differences, the CONTROL rice did not differ significantly from the CRF treatments for the single grain weight. On the field scale, the biomass of aerial parts, panicle and grain weight, and the HI did not significantly differ between the applied top-dressing treatments, but the NUE was significantly higher with CRF_r2_ (*p* < 0.05) (Table 4). No significant differences in grain composition were observed between treatments and the CONTROL. As a result, on average, the quality grain values of the parameters measured for CRF were 1.6% ash, 12.9% humidity, 1.7% lipids, 11.4% protein, 2.5% crude fiber, and 65.8% total carbohydrates.

## 3. Discussion

One of the main challenges for crop development is to increase N fertilization efficiency. In rice, N deficiency reduces growth and generates generalized leaf chlorosis in plants that diminishes photosynthetic processes [9]. As a consequence of N deficiency, N recovery efficiency by plants is usually very low [5]. Nutrient absorption by crops depends on many factors, such as the nutrient type, form and time of application, fertilizer chemical composition, dose, or nutrient availability in soil [9]. In rice, nitrogen fertilization has traditionally been carried out with traditional fertilizers, such as urea and ammonium sulfate [16,39,40]. Under normal aerobic conditions, NH_4_^+^ is easily oxidized to NO_3_^−^ and lost to the atmosphere by denitrification as N_2_ [9]. Usually, flooded rice cultivation allows organic N to be present in soils or not to be oxidized as quickly, so that it is available for plants for longer when applied as NH_4_^+^ during fertilization. Therefore, it is recommended that nitrogen fertilizer in flooded systems such as rice cultivation is applied in an ammoniacal form. One of the main factors that determines the efficiency in N fertilizers applications is proper water management, which helps avoid the presence of aerobic/anaerobic cycles as they accelerate N volatilization and denitrification processes [15,41]. Soils that generally allow these flooding conditions are usually those with a clayey to loamy texture and a good structure, a neutral pH, as well as a compact subsoil horizon to allow good water retention [9].

It is presently considered that using traditional N fertilizers in rice generates a positive benefit/cost ratio. However, the slowdown in the N emissions of fertilizers increases their efficiency by reducing the formulation dose and, consequently, pollution. As mentioned above, the carbohydrate and lignin-based polymers obtained from the waste produced by paper industries have been considered a feasible alternative to synthetics as they are naturally biodegradable polymers that can be obtained at a low cost [23,42,43]. This research work compares in a microscale experiment the effectiveness of a lignosulfonate-based polymer-coated urea fertilizer (CRF) with analogous non polymer-coated urea (DURAMON^®^) and urea. In a second stage, the CRF was compared for its efficacy in the field with ACTIBION^®^ (urea and ammonium-based N-fertilizer). 

No statistically significant differences between the tested fertilizer treatments were observed for most of the growth, physiological, yield, and quality parameters; this was as expected, since all are similar urea-based fertilizers. Nevertheless, some of the obtained results did indicate a (slightly) higher agronomic efficiency of CRF as compared to the other fertilizers. For example, in physiological terms, significant leaf greenness levels were produced in the plants treated with CRF or DURAMON^®^ compared to UREA. These differences could be associated with the beneficial effects of lignosulfonates and humic acids, which can favor micronutrient chelating processes and, consequently, iron availability for plants [44,45]. In photosynthetic terms, the maximum *A* and *gs* values were produced in the plants fertilized with CRF and CRF_r2_, but the CONTROL plant levels were similar. Notwithstanding, the *A/gs* levels at 1566 µmol m^−2^ s^−1^ of the CRF_r2_-fertilized plants were on average significantly higher than for the other treatments. In addition, the *A/E* levels in the CRF treatment were significantly higher compared to DURAMON^®^ and UREA, respectively. These results revealed that the CRF application improved significantly the rice intrinsic water-use efficiency at the photosynthetic level compared to the other treatments. This could benefit nutrient translocation to plants, as increased N foliar levels were found compared to treatments with DURAMON^®^ and UREA at the same dose. N availability in CRF was enhanced by including the urease inhibitor monocarbamida dihidrogenosulfate (MCDHS), but the inhibitor is also present in DURAMON, which lacks coating with the polymer. The application of urease inhibitors in urea-based fertilizers is a practice that is becoming more frequent for its promising results [46,47,48]. In addition, although it is known that N application exerts only synergistic effects on Ca content, it has been observed that CRF application also significantly increases Mg, Fe, and Mn levels. It has been reported that N applications can stimulate root growth and indirectly increase P uptake by increasing the cation exchange capacity of Ca [49]. Higher levels of macro- and micronutrients have also been found in other rice experimentations when applying SCRFs [32,50]. 

At the hormone level, positive effects were observed after applying CRF, which was most likely due to the presence of humic acids. The *ABA* concentration was significantly reduced in those plants fertilized with CRF compared to the treatments performed with UREA and the CONTROL. However, the CRF application significantly improved the concentrations of cytokinins. Indeed, the hormones *iP* and *tZ* significantly increased compared to DURAMON^®^, UREA, and the CONTROL. Using biostimulants as amino acids, humic and/or fulvic acids or extracts of algae can increase the resistance of cultures against abiotic stress and may also influence the hormonal balance of plants [51,52,53]. More specifically, and in agreement with the results of the present work, a recent study demonstrated that humic acids increase the root plasma membrane H^+^-ATPase activity, which in turn, among other effects, mediates an increase in cytokinin concentration in the shoots [54].

The main parameters commonly used to evaluate the efficiency of fertilizers are the biomass and grain yield generated by crops. In fact, the percentage of total N located in panicles when plants reach maturity is around 60–70% [55]. Regarding yield, rice extractions usually come close to 12 kg N per ton of grain [14]. In this research work, the maximum applied doses were 140 NFU to obtain a theoretical yield of 7 tons per hectare on the microscale experiment. On the field scale, no significant differences were obtained between the different fertilizers treatments applied at top dressing. However, rice yields were similar when applying CRF_r2_ (that is, a reduced dose of CRF), as compared to CRF and ACTIBION^®^. This confirmed the results obtained on the microscale in that it is possible to reduce N doses by 20% and obtain the same yield, with a nitrogen-use efficiency (NUE) value higher than in other treatments. 

Rice grain yield is a function of panicles per unit area, the number of spikelets per panicle, grain weight, and spikelet sterility or filled spikelets [9]. Grain weight is mostly dependent on the crop’s genetics and/or variety, and it is not conditioned by N levels [56]. The nutritional status of the plants affects the potential number of grains per panicle during the vegetative growth [9]. On the microscale, the total panicle weight of the CRF treatment was significantly higher compared to CRF_r1_, DURAMON^®^, and UREA. The differences found in panicle weight between treatments were due to the production of significantly more grains with CRF compared to UREA, DURAMON^®^, and CRF reductions. This result was due to the presence of more filled grains because of a reduction in the percentage of aborted seeds. The slow release of N by CRFs allows crops to be able to receive a continuous supply of nutrients and to avoid high initial doses occurring with consequent N losses, possible toxicity, and contamination problems. In this way, slow N release emissions via dose adjustment could reduce the economic and environmental costs of crop production.

## 4. Materials and Methods

### 4.1. Plant Material and Experimental Design

Two experiments were carried out to compare the effectiveness of different fertilizers in rice. (1) The microscale experiment. *Oryza sativa* var. *bahia* was grown outside under ambient conditions but controlling the irrigation regime and leachate sampling at the Valencian Institute of Agrarian Research facilities (Moncada, Spain) from spring to summer 2015. Sowing was performed in cuvettes (150 × 80 × 70 cm; length × width × height, respectively) filled 40–50 cm with farmland soil and placing 25 plants per treatment in five rows with a frame measuring 15 by 15 cm. Flood irrigation was applied with a distilled water depth between 5 and 15 cm; and drainage was performed by a tap located at the bottom of the cuvettes, which was opened only for leachate sampling purposes at the end of the experiment. Agronomic practices such as pesticide treatments or grain protection against bird attack were also performed. (2) The field experiment. *Oryza sativa* var. *bomba* was sown in an experimental plot (1620 m^2^) located in Massanassa (Valencia) with GPS coordinates of 39°24’02.9″ N 0°22’21.3″ W. Sowing was carried out by the direct seeding of 130 kg seeds per ha. Fertilizer treatments were applied on surfaces of 60 × 27 m^2^, and irrigation was applied by continuous flooding. The interference of different fertilizers applied in the field was avoided by excluding the outer rows of each experimental plot when collecting the samples, leaving a security distance of at least 10 m on each side of the plot.

### 4.2. Applied Fertilizers and Treatments

(1) The microscale experiment: Different N fertilizers, developed by Fertinagro Biotech S.L. (Teruel, Spain) were tested and their efficacy was compared: (i) a slow-release fertilizer—DURAMON^®^ (24% nitrogen—0% phosphorus—0% potassium), composed of urea, including a urease inhibitor (monocarbamida dihidrogenosulfate, MCSDH) with no coating (ES 2 204 307 patent); (ii) a controlled-release fertilizer (CRF, hereafter) (24-0-0), based on DURAMON^®^ technology, but also 3% lignosulfonate-coated with natural biostimulants (humic acids); and (iii) a traditional nitrogen fertilizer—UREA (46-0-0). Fertilizers were applied at maximum doses of 140 nitrogen fertilizer units (NFU). CRF and DURAMON^®^ were applied as a basal dressing, and UREA was fractioned as a 70/30 basal/top dressing at the beginning of godson/stem elongation. N dose reductions of 10% were applied as different treatments until 60% of the maximum doses was reached for each experiment (CRF_r1_: 126 NFU; CRF_r2_: 112 NFU; CRF_r3_: 98 NFU; CRF_r4_: 84 NFU). Plants grown without fertilizer addition were used as CONTROL. 

(2) The field experiments: The applied doses (140 NFU) were based on those recommended for rice according to historical yields. The CRFs, applied at 100% (CRF) and 80% (CRF_r2_) of the maximum dose (140 NFU), were compared with the ACTIBION^®^ fertilizer (applying 140 NFU), which is also produced by Fertinagro Biotech S.L. (Teruel, Spain), and it is nutritionally composed (*w/w*) of 22% N, 9% N_ammonia_, 13% N_ureic_, 2.5 % MgO, 20% SO_3_, 1% Fe, 0.05% Mn, 0.05% Zn, and 0.05% Mn and Zn, which were complexed by lignosulfonates; a CONTROL was included as well.

### 4.3. Soil Fertility Characterization

Several soil properties were measured to characterize soil fertility in both experiments [57]. pH and electric conductivity (EC) were determined in a 1/5 (*w/v*) aqueous soil extract by shaking for 2 h, followed by centrifugation at 26,916× *g* for 15 min and filtration. pH was measured with a pH meter (Crison mod. 2001, Barcelona, Spain) and EC was measured with a conductivity meter (Crison micro CM2200, Barcelona, Spain). Total and organic soil C (SOC) and total N (N) were determined by combustion gas chromatography in a Flash EA 1112 Thermo Finnigan (Franklin, MA, USA) elemental analyzer, after eliminating carbonate by acid digestion with HCl. The total nutrient contents (P, K, Ca, Mg, Cu, Fe, K, Mg, Mn, and Zn) were extracted by aqua regia digestion (3:1, *v/v*, HCl/HNO_3_) and determined by Inductively Coupled Plasma Atomic Emission Spectroscopy (ICP-AES) (Thermo Elemental Iris Intrepid II XDL, Franklin, MA, USA). The analyses showed that the rice plants grew on N-poor soil in both experiments (Table 5)

### 4.4. Growth Parameters

Differences in plant growth between fertilizer treatments were determined in the vegetative stage of panicles swelling and the booting stage for the microscale experiment. The studied growth parameters were total length (cm), primary stem length (cm), tillers number, leaf weight (g), and foliar area (cm^2^) (using a LI-3100C area meter—LI-COR^®^, Nebraska, USA). Some plant material was weighed before being dried at 65 °C until a constant mass was obtained to calculate the dry mass percentage. Relative water content was calculated as RWC (%) = (FW–DW) / (TW–DW) × 100, where FW is fresh mass, TW is turgid mass after saturating leaves with water at 4 °C in the dark, and DW is dry mass after oven-drying leaves at 65 °C for 72 h [58].

### 4.5. Leaf Gas Exchange and Photosynthetic Parameters

Gas exchange measurements were taken at noon in five plants per treatment, using a portable infrared gas analyzer LCpro-SD, which was equipped with a PLU5 LED light unit (ADC BioScientific Ltd, Hoddesdon, UK) for the microscale experiment. The selected flag leaves in rice in the booting stage were analyzed with a broadleaf chamber (6.25 cm^2^) to determine the following parameters: stomatal conductance (*gs*) (expressed as mmol m^−2^ s^−1^), net photosynthetic rate (*A*) (µmol m^−2^ s^−1^), transpiration (*E*) (mol m^−2^ s^−1^), and intercellular CO_2_ concentration (*Ci*) (µmol mol^-1^) under ambient CO_2_, temperature and relative humidity conditions. They were recorded using photosynthetically active radiations (PAR) ranging from 400 to 1800 µmol m^−2^ s^−1^. Water-use efficiency (WUE) and intrinsic WUE were calculated as the ratio between *A/gs* and *A/E*, which was expressed as µmol (CO_2_ assimilated) mol^−1^ (H_2_O transpired). Leaf greenness was measured by a SPAD-502 Chlorophyll meter (Konica-Minolta, Osaka, Japan) [59]. The effective quantum yield of photosystem II electron transport (ΦPSII), which represents the electron transport efficiency between photosystems within light-adapted leaves, was checked using a leaf fluorometer (Fluorpen FP100, Photos System Instrument, Drásov, Czech Republic). Both parameters were measured in a minimum of 25 leaves, as indicated for leaf gas exchange.

### 4.6. Foliar Nutrient Analysis

Foliar analyses for the microscale experiment were performed from fresh samples collected at the same vegetative stage indicated for the study of growth parameters on July 7. Samples were composed of a pool of minimum 10 flag leaves, which were taken randomly from different plants for each treatment. Four pool-replicates per treatment and culture were collected, and these were kept at −20 °C until their biochemical analyses were performed. The compositions in macro- (N, P, K, Ca, and Mg) and micronutrients (Fe, Cu, Mn, Zn, B, and Mo) were determined by Inductively Coupled Plasma Optical Emission Spectrometry (ICP-OES). N content was estimated by an N-Pen N 100 apparatus (Photon System Instruments, Drásov, Czech Republic).

### 4.7. Hormone Activity

The samples used for the foliar nutrient analysis were also used to determine the activity of different hormones related to plant development, such as indoleacetic acid (*IAA*), jasmonic acid (*JA*), salicylic acid (*SA*), abscisic acid (*ABA*), and cytokinins (*CK*), including isopentenyl adenine (*iP*), t-zeatin (*tZ*), and dihydrozeatin (*DHZ*). Analyses were performed by the Plant Hormone Quantifying Service (IBMCP-UPV) in a Thermo Scientific™ Q Exactive™ Hybrid Quadrupole-Orbitrap Mass Spectrometer (LC-MS/MS HR). Hormone content was expressed as ng g^-1^ of leaf dry weight.

### 4.8. Yield and Cereal Grain Composition

Once grain ripening was reached in the microscale (Oct 9) and field (Sept 13) experiments, the remaining plants were harvested, and growth parameters and grain yield were determined. The growth parameters were the total fresh and dry weights of aerial parts (g), primary stem length (cm), and tillers number. Yield was quantified by measuring the following parameters: total panicle weight (g), panicle length (cm), panicles number, mean panicle weight (g), weight of 1000 grains (g), grain number, and the nitrogen-use efficiency (NUE) (kg kg^−1^), which was calculated as the quotient between the grain yield of the fertilized area and the quantity of N applied as fertilizer. To evaluate yields, at the microscale, 20 plants were taken per treatment. On the field scale, the growth and yield parameters were studied 82 days after applying the fertilizers, including the biomass of aerial parts, panicles weight, and grain weight expressed as tons per hectare (t ha^−1^). A pool of all plants contained in one meter square by four replications for each treatment was selected randomly avoiding plot borders. Field-yield results were confirmed by full mechanical harvest and grain weighing. The harvest index (HI) was calculated as the grain weight/biomass of aerial parts. Different quality parameters were also measured in grain, based on food quality analysis methods (Commission Regulation EC Nº 152/2009 of January 27): humidity (gravimetric by drying in an oven at 130 °C), ashes (gravimetric by incineration at 550 °C), lipids (extraction without hydrolysis in Soxtec Avanti–Foss), protein (Kjeldahl method using Foss automatic distillation equipment), crude fiber (gravimetric), and total carbohydrates (volumetric using Luff Schoorl reagent). Analyses were carried out by the Valencia’s Agrifood Laboratory (Burjassot, Spain).

### 4.9. Statistics

The statistical differences between the fertilizer treatments for the different measured parameters were processed by analysis of variance (ANOVA) at the *p* < 0.05 confidence level. Before the analysis of variance, the data requirements of normality and homogeneity of variances were checked according to Levene’s and Shapiro–Wilk tests. When the null ANOVA hypothesis was rejected, post hoc comparisons were made to establish any possible statistical differences between the different treatments applied using Tukey’s test. The statistical SPSS v.16 software program (SPSS, Inc., Chicago, USA) was used to perform the analysis.

## 5. Conclusions

In this research work, a lignin-coated, controlled-release fertilizer, containing humic substances developed by Fertinagro Biotech S.L. (Teruel, Spain), was compared in its efficacy with other fertilizers also having a ureic base but not being polymeric-coated. In the microscale experiment, increased nitrogen and water-use efficiency and yield were found when applying this new CRF compared to DURAMON^®^ and UREA. On the field scale, CRF top-dressing applications gave similar results to ACTIBION^®^. It was possible to reduce CRF doses by 20% to obtain the same yields as with the other fertilizers. The possibility of using CRFs in extensive cropping with lignin-based wastes is new in rice production, and it is possible to gain higher yields without increasing costs, as well as to facilitate crop management. The controlled emissions of N performed by applying this CRF and its biodegradability reduce contamination. In short, this CRF is environmentally friendlier than synthetic or traditional fertilizers. Additional field studies are needed to perform a more precise cost/benefit assessment of the use of CRF in rice-flooded cultivation. Nevertheless, even though the differences with the other tested fertilizers were modest, the results presented here suggest that this CRF will provide yields at least similar to traditional fertilizers, improving the agronomic management of the crop, and minimizing the environmental impact of N rice fertilization.

## Figures and Tables

**Table 1:** Effects of the fertilizer treatments, in the microscale experiment, on the effective quantum yield of photosystem II (ΦPSII), leaf greenness, and N content in the phenological stage of panicles swelling (booting stage) (*n* = 20 leaves per treatment). Comparisons per plant for each treatment were also performed measuring the total fresh weight, dry weight, total length, primary stem length, tillers number, leaf weight, leaf relative water content, and total foliar area (values are means ± SD; *n* = 5 plants). Treatments: controlled-release fertilizer (CRF), analogous non polymer-coated urea (DURAMON^®^), and UREA: 140 nitrogen fertilizer units (NFU); CRF_r1_: 126 NFU; CRF_r2_: 112 NFU; CRF_r3_: 98 NFU; CRF_r4_: 84 NFU; CONTROL (no fertilizer applied).

Parameters	CRF	CRF_r1_	CRF_r2_	CRF_r3_	CRF_r4_	DURAMON^®^	UREA	CONTROL
ΦPSII	0.71 ± 0.05 b	0.69 ± 0.07 b	0.70 ± 0.07 b	0.69 ± 0.06 b	0.67 ± 0.09 ab	0.71 ± 0.07 b	0.69 ± 0.04 b	0.62 ± 0.02 a
Leaf greenness content (SPAD units)	51.23 ± 8.07 b	47.22 ± 6.04 ab	47.80 ± 5.43 ab	46.88 ± 3.48 ab	45.76 ± 2.14 a	48.18 ± 4.75 ab	45.01 ± 3.65 a	44.58 ± 6.54 a
N content (%)	8.23 ± 1.02 c	8.37 ± 0.51 c	7.50 ± 0.66 bc	7.30 ± 0.62 bc	6.75 ± 0.68 b	7.00 ± 1.68 bc	6.55 ± 0.84 b	4.23 ± 1.18 a
Total fresh weight (aerial part) (g)	107.2 ± 26.6 ab	134.8 ± 21.2 b	135.2 ± 36.6 b	127.8 ± 34.5 b	129.6 ± 25.9 b	128.0 ± 24.5 b	115.6 ± 26.4 ab	64.4 ± 6.2 a
Dry weight (aerial part) (%)	23.90 ± 1.20 a	27.10 ± 1.60 ab	23.60 ± 0.70 a	28.50 ± 2.60 b	27.60 ± 2.20 ab	28.30 ± 3.10 b	30.10 ± 1.90 b	29.20 ± 1.80 b
Total length (cm)	69.70 ± 1.80 b	71.50 ± 2.20 b	67.10 ± 5.30 ab	71.70 ± 3.20 b	67.20 ± 30 ab	70.80 ± 3.30 b	70.40 ± 2.40 b	62.30 ± 2.40 a
Primary stem length (cm)	46.00 ± 2.90 a	45.70 ± 2.80 a	46.30 ± 2.80 a	43.60 ± 2.70 a	46.20 ± 2.80 a	41.50 ± 7.60 a	43.60 ± 1.90 a	41.60 ± 6.90 a
Tillers number	14.00 ± 3.10 ab	13.40 ± 2.50 ab	13.20 ± 3.10 ab	11.80 ± 4.50 ab	12.80 ± 1.30 ab	15.80 ± 3.10 b	13.40 ± 40 ab	7.80 ± 1.90 a
Leaf weight (g)	0.13 ± 0.01 ab	0.16 ± 0.01 c	0.15 ± 0.01 bc	0.15 ± 0.01 c	0.13 ± 0.01 abc	0.15 ± 0.01 abc	0.15 ± 0.01 abc	0.13 ± 0.02 a
Leaf RWC (%)	92.20 ± 4.70 b	91.50 ± 3.80 b	93.90 ± 3.20 b	92.20 ± 2.10 b	83.30 ± 2.90 ab	92.60 ± 2.80 b	92.00 ± 2.50 b	82.80 ± 8.00 a
Total foliar area (cm^2^)	10.80 ± 2.50 b	12.50 ± 1.80 b	12.00 ± 3.30 b	11.10 ± 3.80 b	10.60 ± 2.10 b	12.10 ± 2.90 b	11.60 ± 3.70 b	3.40 ± 1.30 a

Different letters in the same row indicate significant statistical differences (Tukey’s test, *p* < 0.05).

**Table 2:** Hormone activity comparison between the applied fertilizer treatments (CRF and their reduction to 80% CRF_r2_, DURAMON^®^, and UREA) compared to the CONTROL (no fertilizer applied) in the microscale experiment. The quantified hormones were indoleacetic acid (*IAA*), jasmonic acid (*JA*), salicylic acid (*SA*), abscisic acid (*ABA*), and cytokinins, including isopentenyl adenine (*iP*), and t-zeatin (*tZ*). Values are means ± SD (*n* = 4) at the end of the culture and expressed as ng hormone g^−1^ FW leaves.

	CRF	CRF_r2_	DURAMON^®^	UREA	CONTROL
*IAA*	1.29 ± 0.04 a	1.83 ± 0.07 a	1.41 ± 0.06 a	1.30 ± 0.06 a	1.44 ± 0.06 a
*JA*	0.29 ± 0.06 a	0.42 ± 0.05 b	0.55 ± 0.06 c	0.85 ± 0.10 d	0.27 ± 0.08 a
*SA*	5499.5 ± 1758.2 a	6841.8 ± 928.0 ab	7841.6 ± 959.5 b	7657.9 ± 714.3 b	7255.2 ± 486.0 b
*ABA*	15.27 ± 1.30 a	14.43 ± 1.13 a	19.48 ± 0.34 a	19.91 ± 0.71 ab	22.54 ± 1.12 b
*iP*	0.18 ± 0.07 c	0.05 ± 0.01 b	0.02 ± 0.01 a	0.03 ± 0.01 a	0.02 ± 0.01 a
*tZ*	0.47 ± 0.11 c	0.46 ± 0.07 c	0.10 ± 0.02 a	0.24 ± 0.04 b	0.20 ± 0.01 ab

Different letters in the same row indicate significant statistical differences (Tukey’s test, *p* < 0.05).

**Table 3:** Comparison of the growth and yield parameters per plant between the applied fertilizer treatments (CRF and their reductions CRF_r1_–CRF_r4_, DURAMON^®^, and UREA) compared to the control in the microscale experiment at the end of culture (CRF, DURAMON^®^, and UREA: 140 NFU; CRF_r1_: 126 NFU; CRFr_2_: 112 NFU; CRF_r3_: 98 NFU; CRF_r4_: 84 NFU); CONTROL (no fertilizer applied). Values are presented as means ± SD (*n* = 20 plants).

Parameters	CRF	CRF_r1_	CRF_r2_	CRF_r3_	CRF_r4_	DURAMON^®^	UREA	CONTROL
Total fresh weight (aerial part *) (g)	149.9 ± 46.0 c	147.3 ± 41.9 bc	122.6 ± 43.8 b	152.6 ± 44.1 c	128.5 ± 46.8 bc	154.7 ± 43.4 c	139.1 ± 42.4 bc	88.4 ± 28.8 a
Dry weight (aerial part *) (%)	18.4 ± 3.5 bc	27.1 ± 6.9 d	19.5 ± 3.1 b	19.8 ± 3.3 c	17.7 ± 2.3 b	19.9 ± 1.9 c	19.9 ± 3.6 bc	19.8 ± 2.5 a
Primary stem length (cm)	56.8 ± 2.6 d	57.9 ± 2.7 d	54.3 ± 3.4 c	54.7 ± 3.2 c	53.1 ± 3.2 bc	53.9 ± 3.4 bc	52.4 ± 3.7 b	47.8 ± 1.9 a
Tillers number	14.4 ± 3.7 abc	15.9 ± 7.5 abc	14.1 ± 5.7 ab	16.1 ± 6.2 bc	15.1 ± 6.9 abc	19.0 ± 7.5 c	15.8 ± 6.9 bc	12.0 ± 4.9 a
Total panicle weight (g)	51.5 ± 16.8 c	38.8 ± 11.5 b	35.9 ± 12.8 b	38.0 ± 9.2 b	37.7 ± 11.5 b	37.1 ± 7.8 b	33.1 ± 10.3 b	21.2 ± 7.3 a
Panicle length (cm)	12.8 ± 0.6 bc	11.9 ± 1.0 ab	11.9 ± 0.7 ab	11.8 ± 0.4 ab	12.7 ± 0.6 bc	12.7 ± 0.7 bc	13.6 ± 1.2 c	11.0 ± 0.9 a
Panicle number	12.9 ± 3.0 ab	14.8 ± 6.8 b	12.9 ± 4.8 ab	14.1 ± 4.2 b	14.1 ± 4.9 b	14.3 ± 4.4 b	11.1 ± 3.7 a	10.3 ± 4.2 a
Mean panicle weight (g)	4.0 ± 0.8 c	3.1 ± 1.4 c	2.9 ± 0.9 c	2.8 ± 0.8 bc	2.7 ± 0.4 a	2.7 ± 0.6 ab	3.1 ± 0.9 bc	2.3 ± 1.0 bc
Weight of 1000 grains (g)	31.0 ± 1.5 bc	28.6 ± 1.5 ab	30.3 ± 1.9 bc	29.6 ± 1.4 abc	30.1 ± 0.6 abc	27.6 ± 3.8 a	29.8 ± 1.7 abc	31.4 ± 1.7 c
Grain number × 0.01	16.6 ± 5.4 c	13.6 ± 4.0 b	11.8 ± 4.2 b	12.8 ± 3.1 b	12.5 ± 3.8 b	13.5 ± 2.8 b	11.1 ± 3.5 b	6.7 ± 2.3 a
Grain yield (g)	50.5 ± 16.5 c	38.1 ± 11.3 b	35.2 ± 12.6 b	37.2 ± 9.0 b	36.8 ± 11.3 b	36.4 ± 7.7 b	32.3 ± 10.1 b	20.6 ± 7.2 a

Different letters in the same row indicate significant statistical differences (Tukey’s test, *p* < 0.05). * Total tillers without including panicles.

**Table 4:** Field harvest comparison of the growth parameters and grain yield of *Oryza sativa* var. *bomba* between the different fertilizer treatments applied: CRF, CRF_r2_, and ACTIBION^®^ (CRF and ACTIBION^®^: 140 NFU; CRF_r2_: 112 NFU) compared to the CONTROL (no fertilizer applied). Values are means ± SD, *n* = 4 (pools of plants in 1 m^2^) at the end of the culture, corresponding to 82 days after applying the fertilizers.

Parameters	CRF	CRF_r2_	ACTIBION^®^	CONTROL
Biomass of the aerial part (t ha^−1^)	18.31 ± 2.58 ab	19.80 ± 4.00 ab	21.23 ± 3.78 b	15.14 ± 2.82 a
Panicle weight (t ha^−1^)	5.85 ± 0.71 b	6.85 ± 1.69 b	6.32 ± 0.83 b	3.74 ± 0.98 a
Grain weight (t ha^−1^)	5.44 ± 0.78 b	6.31 ± 1.43 b	5.81 ± 0.61 b	3.40 ± 0.88 a
Nitrogen Use Efficiency (kg kg^−1^ N)	38.89 ± 5.60 a	56.34 ± 12.74 b	41.54 ± 4.33 a	-
Harvest Index	0.48 ± 0.02 a	0.51 ± 0.03 a	0.49 ± 0.03 a	0.46 ± 0.07 a

Different letters in the same row indicate significant statistical differences (Tukey’s test, *p* < 0.05).

**Table 5:** Fertility of the soil used in the experimental analysis from the first 15 cm of soil surface. Data on total nitrogen (N), total carbon (C), and organic carbon (CO) and other macro- and micronutrients are shown. Values are means ± SD (*n* = 5) at the beginning of the experiment.

Parameters	Mean ± SD	
	Microscale	Field
Total nitrogen (g 100 g^−1^)	0.12 ± 0.04	0.18 ± 0.02
Total carbon (g 100 g^−1^)	7.09 ± 0.08	6.74 ± 0.12
Organic carbon (g 100 g^−1^)	1.13 ± 0.18	2.03 ± 0.11
pH	8.5 ± 0.03	7.89 ± 0.04
EC (µS cm^−1^)	470.33 ± 34.12	645.25 ± 111.12
P (g 100 g^−1^)	0.051 ± 0.004	0.083 ± 0.007
K (g 100 g^−1^)	1.46 ± 0.12	0.81 ± 0.07
Mg (g 100 g^−1^)	1.87 ± 0.14	0.058 ± 0.04
Ca (g 100 g^−1^)	15.39 ± 1.06	11.77 ± 1.10
Fe (g 100 g^−1^)	2.43 ± 0.02	1.46 ± 0.07
Cu (mg kg^−1^)	32.54 ± 3.39	51.83 ± 2.96
Mn (mg kg^−1^)	339.57 ± 27.12	170.61 ± 11.25
Zn (mg kg^−1^)	43.19 ± 4.14	97.74 ± 6.45
